# Tuning of Hydrogel Architectures by Ionotropic Gelation in Microfluidics: Beyond Batch Processing to Multimodal Diagnostics

**DOI:** 10.3390/biomedicines9111551

**Published:** 2021-10-27

**Authors:** Alessio Smeraldo, Alfonso Maria Ponsiglione, Paolo Antonio Netti, Enza Torino

**Affiliations:** 1 Department of Chemical, Materials and Production Engineering, University of Naples Federico II, Piazzale Tecchio 80, 80125 Naples, Italy; alessio.smeraldo@unina.it (A.S.); alfonsomaria.ponsiglione@unina.it (A.M.P.); paoloantonio.netti@unina.it (P.A.N.); 2 Center for Advanced Biomaterials for Health Care—CABHC, Istituto Italiano di Tecnologia, IIT@CRIB, Largo Barsanti e Matteucci 53, 80125 Naples, Italy; 3 Interdisciplinary Research Center on Biomaterials—CRIB, Piazzale Tecchio 80, 80125 Naples, Italy

**Keywords:** microfluidics, ionotropic gelation, hydrogel, Hydrodenticity, multimodal imaging

## Abstract

Microfluidics is emerging as a promising tool to control physicochemical properties of nanoparticles and to accelerate clinical translation. Indeed, microfluidic-based techniques offer more advantages in nanomedicine over batch processes, allowing fine-tuning of process parameters. In particular, the use of microfluidics to produce nanoparticles has paved the way for the development of nano-scaled structures for improved detection and treatment of several diseases. Here, ionotropic gelation is implemented in a custom-designed microfluidic chip to produce different nanoarchitectures based on chitosan-hyaluronic acid polymers. The selected biomaterials provide biocompatibility, biodegradability and non-toxic properties to the formulation, making it promising for nanomedicine applications. Furthermore, results show that morphological structures can be tuned through microfluidics by controlling the flow rates. Aside from the nanostructures, the ability to encapsulate gadolinium contrast agent for magnetic resonance imaging and a dye for optical imaging is demonstrated. In conclusion, the polymer nanoparticles here designed revealed the dual capability of enhancing the relaxometric properties of gadolinium by attaining Hydrodenticity and serving as a promising nanocarrier for multimodal imaging applications.

## 1. Introduction

Nanostructured materials have attracted considerable interest over the last few years due to their tunable multifunctional properties [1,2,3,4]. Among them, polymer nanoparticles (NPs) are giving promising results for improving imaging techniques and therapeutic approaches [5,6]. In addition to the well-known general advantages of such nanovectors [7], it has been proved that polymer NPs, particularly hydrogels, can be designed to enhance imaging performances, increase specificity, and potentially reduce the fast clearance of drugs and diagnostic agents from the bloodstream [8,9,10,11,12].

Among the polymers mainly used for medical applications [13,14,15,16,17], hyaluronic acid (HA) and its derivatives have been investigated for the development of nanomedicine [18,19,20,21]. HA has also been used in combination with Chitosan (CS) for the synthesis of drug delivery systems [19,22,23]. For example, Chen and co-workers [24] have reported a yolk–shell structure based on an up-conversion luminescent in a silica shell with an HA/CS coating for pH-triggered drug release. Remarkable results have been achieved by Courant et al. [9], who have randomly coprecipitated HA and CS to obtain high-relaxivity gadolinium-based nanoparticles for magnetic resonance imaging (MRI).

More recently, Vecchione et al. [10,25] produced a CS/HA core–shell nanoarchitecture for multimodal imaging through a complex coacervation process driven by temperature and high-pressure homogenization. Such a nanostructure, designed to co-encapsulate a clinically relevant contrast agent (CA) for MRI and a tracer for optical imaging, has been then decorated with the peptide pA20–36 to selectively target B-cell lymphoma cells and successfully tested in a murine model for in vivo theranostic applications [26].

As shown in their previous works [11,27,28,29,30,31], these authors demonstrated that the proper control of the structural properties of polymer-based nanohydrogels, e.g., by tuning their crosslinking density, mesh size and hydrophilicity, can impact on the relaxometric properties of the MRI CA entrapped in the polymer network enhancing its relaxivity, i.e., the measure of the efficacy of the CA itself. Indeed, Russo et al. explained that the boosting in the relaxivity is achieved when a complex equilibrium is reached between the water osmotic pressure, the elastodynamic forces of the polymer chains and the hydration degree of the CA [11,31]. This equilibrium responsible for the relaxation enhancement, previously defined as the novel concept of Hydrodenticity [31], can be attained, under specific conditions, by controlling the process parameters used to produce CA-loaded nanostructures.

As described above, the structural properties of the polymer network emerge as the leading parameters to give multiple functionalities to the nanostructures. In this regard, the combined use of two or more different polymers allows a further tuning of the structural properties [32,33,34] and it is worth highlighting that CS and HA have been proven to have a huge potential in the application to this field [35,36,37,38,39]. Among the most-used methods to combine CS and HA into multifunctional nanovectors with desired physicochemical and morphological properties [17,40,41,42,43,44], ionotropic gelation has proven to be a complex but promising way to synthesize CS–HA nanoparticles. It is based on the interaction of a cation (or an anion) with one or more ionic polymers to generate a highly inter or intra crosslinked structure. Despite the widespread use of ionotropic gelation, it has been reported that fine control over the final product’s features is difficult to achieve through the traditional batch protocols [45,46], whose main drawbacks lies in the poor control of mixing and separation of particles, resulting in polydispersity and batch-to-batch variations [47], thus limiting the translation of CS–HA-based architecture in preclinical and clinical practice.

In this perspective, the design of processes based on microfluidics already proved to have the ability to overcome this issue, improving the synthesis of nanoparticles and accelerating their transition to clinical evaluation [48,49,50,51,52,53,54]. Reactions in microfluidic devices are carried out with a low amount of fluid within small channels [54,55,56,57]. It enables fine control and manipulation of fluids and their interfaces, and rapid and uniform heat and mass transfer thanks to the established laminar flow [58,59,60,61].

In particular, microfluidics is a promising and effective tool for the rational design of polymer NPs as imaging probes and drug delivery systems [62,63]. As shown in recent studies conducted by Russo et al. [64,65], the microfluidic hydrodynamic flow focusing (HFF) approach allows fine tuning of the structural characteristics of HA-based nanohydrogels and, in the presence of an MRI CA, permits the attainment of the above-mentioned Hydrodenticity, thereby increasing the relaxometric properties of the CA entrapped within the nanostructure.

This work proposes for the first time the exploitation of ionotropic gelation by microfluidics applied to the design and production of CS–HA NPs. Grounding on the acquired know-how in microfluidic synthesis of polymer nanoparticles by hydrodynamic flow focusing, we aim to demonstrate the power of matching ionotropic gelation with microfluidics in tuning the morphology of the complex architectures. For this purpose, we used a custom-designed microfluidic chip with a specific geometry tailored to achieve the desired hydrodynamic flow-focusing conditions and coupling of the compounds. Furthermore, some preliminary results about the capability of the obtained architectures to encapsulate simultaneously both MRI CAs and optical tracers in a one-step process are reported. The Hydrodenticity behavior of the nanostructures is also evaluated.

## 2. Materials and Methods

### 2.1. Materials

Hyaluronic acid (HA) with a molecular weight (Mw) of 50,000 Da was purchased from CreativePEGWorks (Chapel Hill, NC, USA). Chitosan (CS) with a low molecular weight of 50,000 Da and sodium tripolyphosphate (TPP) with a Mw of 367.86 Da produced by Sigma-Aldrich were chosen in this study. Acetone (Sigma-Aldrich, St. Louis, MO, USA) is used for collection and dialysis, while ethanol (Carlo Erba, Milan, Italy) is employed in the successive step to change sample collection from acetone to water. Commercially available Gd-DTPA (Sigma-Aldrich, St. Louis, MO, USA) with a Mw of 547.57 Da is used since it is a well-known, low-risk CA. Atto-488 (Mw = 804 Da; λ = 480–515 nm) was purchased by Sigma-Aldrich. Milli-Q water (Milli-Q Plus, Q-POD®, Merck KGaA, Darmstadt, Germany) is used to prepare solutions and dialysis.

### 2.2. Microfluidic Platform

**Figure 1:**
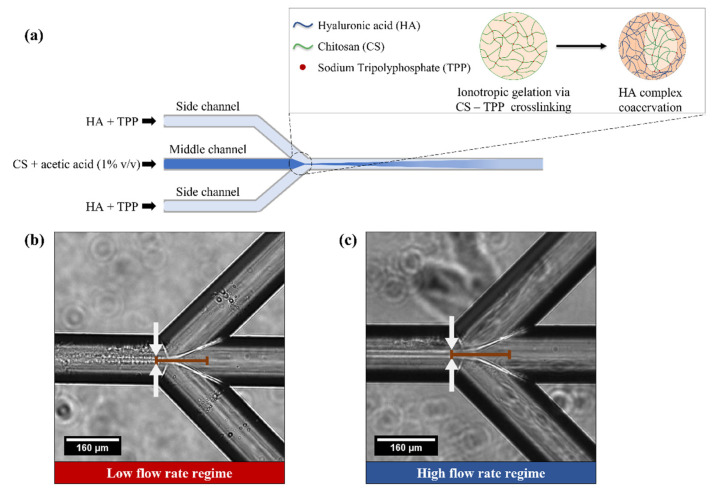
(**a**) Schematic representation of the hydrodynamic flow focusing within microfluidic chip. Middle and side streams lead to NPs formation through ionotropic gelation via CS-TPP crosslinking followed by HA–CS complex coacervation; (**b**,**c**) Optical image of the microfluidic device showing the hydrodynamic flow focusing at (**b**), low flow rate regime (middle channel flow rate < 1 μL/min) and (**c**) high flow rate regime (middle channel flow rate ≥ 1 μL/min). White arrows in both images indicate the width of the focusing stream, while the black line along the middle channel represents its elongation at the chip junction.

The microfluidic platform is composed of a system of three syringes (5 mL PTFE PEEK tubing connector, SETonic GmbH, Ilmenau, Germany), each controlled by a low-pressure syringe pump (Low Pressure Syringe Pump neMESYS 290N by CETONI, Korbußen, Germany). Syringes are connected to the microfluidic chip through PTFE tubing and connectors and are equipped with 2-way in-line valves to manually open and close the line for each syringe. All reactions and processes are conducted in the main body of a custom-made quartz glass microfluidic chip, designed to obtain a hydrodynamic flow focusing at the channel junction. All channels of the microfluidic chip have the same cross-section of 160 × 150 μm. Fluid from the outlet is collected in a glass Petri dish prefilled with water or acetone. A schematic representation of the process is discussed in the results section and displayed in Figure 1.

### 2.3. Production of CS-HA Nanoparticles

Middle and side streams include two different polymers that lead to NP formation through ionotropic gelation via CS-TPP crosslinking followed by HA-CS complex coacervation. The starting step consists of the preparation of polycationic and polyanionic solutions. Components are withdrawn from previously prepared stocks at 0.2% *w*/*v*. The first one is obtained by mixing CS (concentration range from 0.00625 to 0.2% *w*/*v*) in an acetic acid buffer (1% *v*/*v*), while the second one is obtained by dissolving HA (concentration range from 0.002 to 0.008% *w*/*v*) and TPP (concentration range from 0.003 to 0.012% *w*/*v*) in water. Both solutions are stirred at 300 rpm for 30 min. Polycationic solution (CS) is pushed in the middle channel, while polyanionic solution (HA + TPP) is pushed in the two side channels. Sample is collected in Petri dishes filled with acetone and covered with aluminum foil to limit solvent evaporation. After each usage, the chip is repeatedly washed with water and 1% *v*/*v* acetic acid aqueous solution. Possible residuals of the precipitated materials within the channels, that cannot be removed by washing the chip, are then cleaned by immersing the chip in a piranha solution composed of ¼ nitric acid and ¾ sulfuric acid overnight.

### 2.4. Physico-Chemical and Morphological characterization of CS-HA Nanoparticles

NP morphological and structural surface features are analyzed with a scanning electron microscope (SEM, Ultraplus Field Emission, Carl Zeiss, Oberkochen, Germany). Examined samples are dropped on glass directly from the microfluidic platform outlet or filtered, after collection and dialysis, on a membrane of 50 nm pore size. Nanoparticles are coated with 5.5 nm Au or Pt/Pd prior observation. Another characterization is carried out with a TEM (Tecnai FEI^®^ transmission electron microscope, Hillsboro, OR, USA) that allows examining NPs’ internal features. Samples are collected on Formvar/Carbon 200 mesh Cu Agar^®^ small net from platform outlet or dropping off 20–50 μL of solution on it, before or after dialysis.

### 2.5. Gd-DTPA Loading and Evaluation of the Encapsulation Efficiency

Induced Coupled Plasma Mass Spectrometry (ICP-MS) NexION 350 by Perkin Elmer Inc. (Waltham, MA, USA) is used to assess the concentration of Gd-DTPA loaded within the NPs. A suspension of CS-HA nanoparticles in deionized water at a concentration of 250,000 particles/mL without dissolution is injected. All data are collected and processed using the Syngistix NanoApplication (Module PerkinElmer Inc., Waltham, MA, USA). Gd is measured at m/z 157 using a 100 μs dwell time with no settling time. Successively, results are compared to the known initially added amount of Gd-DPTA to obtain an estimate of the encapsulation efficiency.

### 2.6. In Vitro MRI

Analyses with Minispec mq60 BRUKER benchtop relaxometer (Bruker Corporation, Billerica, MA, USA, magnetic field strength: 1.41 T) are performed to evaluate the relaxation times. The sample is loaded within a glass tube and then placed into the NMR probe for about 15 min for thermal equilibration. The Free Induction Decay sequence (FID) is used to evaluate the best value of the gain to control the saturation of the signal. Longitudinal relaxation times, $T_{1}$, are determined by saturation recovery pulse sequence. The relaxation time distribution is obtained by CONTIN Algorithm [66].

### 2.7. Spectrofluorometer

Atto-488 amount is detected through a spectrofluorimetric reading (EnSpire Multimode Plate Reader, PerkinElmer Inc, Waltham, MA, USA) that provides information regarding fluorophore concentration in the sample in a range of 0–250 pmol/mL.

### 2.8. Preliminary In-Vitro Cell Tests

Cytotoxicity studies on human brain glioblastoma astrocytoma cells (U87-MG) are performed to preliminarily assess NP biocompatibility. U87 MG cells are seeded in 96-well plates (5 × 10^4^ cells/well) and allowed to adhere for 24 h in free medium (DMEM, 1% penicillin/streptomycin and 1% L-glutamine). Adherent cells are then incubated with medium supplied with NPs (22 µg/mL) or free medium as negative control. Cells are checked for viability at 8 or 24 h by means of an MTT test.

## 3. Results

### 3.1. Ionotropic Gelation Controlled by Hydrodynamic Flow Focusing for Production CS-HA Nanostructures

As previously reported [11,67], hydrodynamic flow focusing (HFF) is obtained when fluids with different velocities flow side by side into microfluidic channels. A middle stream is squeezed between two adjacent streams working at a higher flow rate. The ratio between the flow rate of the middle channel (µL/min) and the flow rate of only one of the side channels (µL/min), namely Flow Rate Ratio (FR^2^), is calculated as follows [11]:

$${\mathrm{FR}}^{2}=\frac{Flow\;Rate{|}_{middle\;channel}}{Flow\;Rate{|}_{side\;channel}}$$

Moreover, HFF has been applied to different processes, among them flow focused nanoprecipitation, where the lower the FR^2^, the narrower the hydrodynamic flow focusing, whose width is strictly linked to mutual mixing and diffusion phenomena of the flows and responsible for the thermodynamic process [68]. Indeed, the relationship between FR^2^ and the mixing time is modeled according to the following equation:

$$\tau_{mix}∼\frac{w_{f}^{2}}{4D}\approx \frac{w^{2}}{9D}\;\frac{1}{{\left(1+1/{\mathrm{FR}}^{2}\right)}^{2}}$$

where $w_{f}$ is the width of the hydrodynamic flow focusing, *w* represents the channel width and *D* the diffusion coefficient of the solvent [55].

Several studies conducted on flow focused nanoprecipitation also demonstrated that at a low Reynolds number, laminar flow is achieved within the microchannels, enabling proper supersaturation conditions and homogenous particle formation kinetics [55,69,70].

Here, we take advantage of the theoretical knowledge of HFF to induce ionotropic gelation within the microchannel and control the morphology of the nanostructures by tuning the process parameters, mainly flow rates, FR^2^, concentration and ratio of the reagents.

Generally, in ionotropic gelation, TPP binds to the charged amino groups of CS, allowing the formation of a three-dimensional network of the ionic crosslinked moiety [10]. Usually, in the ionic gelation method, chitosan is dissolved in an aqueous acidic solution to obtain the cation of Chitosan. This solution is then added dropwise continuously based on the capability of polyelectrolytes to traverse links in the presence of counter ions to form nanoparticles [71,72,73]. In our system, HA and CS are chosen as oppositely charged polyelectrolytes able to bind together, stabilized by the presence of TPP. Chitosan undergoes ionic gelation and precipitates to form spherical particles due to the complexation between oppositely charged species.

In our system, a water solution HA and TPP flow through the side channels, while a 1% acetic acid aqueous solution of CS is injected through the middle channel of the microfluidic chip. A schematic representation of the process to produce the NPs is shown in Figure 1.

In the proposed set up (Figure 1a), the chitosan is dissolved in an aqueous acidic solution to obtain the cation of chitosan, while the kinetics of the gelation is controlled by hydrodynamic flow focusing through the lateral injection of HA and TPP at different flow rates. This fine control of the flow rate ratio tunes the mixing time and dosage among the reagents.

NP formation is achieved through the partial precipitation of the chitosan in water and ionotropic gelation via CS-TPP-HA complex coacervation along the middle stream.

A similar approach that takes advantage of the mutual diffusion and precipitation of the components has already been published by our group using an emulsion-based batch approach and it is proposed here, for the first time, in a continuous mode using microfluidics to avoid polydispersity and improve purification and control of the structural properties [10,25,26].

In this microfluidics approach, different parameters have been tested using this configuration (as reported in Table S1 of the Supplementary Materials), with particular attention paid to the following: (i) FR^2^ (ranging from 0.05 to 0.5); (ii) flow rates (ranging from 0.2 to 20 μL/min for the middle channel and from 0.5 to 100 μL/min for side channels); (iii) CS:HA weight ratio calculated at the chip junction (ranging from 0.0781 to 6.25). The rationale for calculating the CS:HA weight ratio within the microchip is explained in Equation S1 and displayed in Figure S1 of the Supplementary Materials.

### 3.2. Identification of Operating Regimes and Fluidodynamic Threshold for the Experimental Campaign

**Figure 2:**
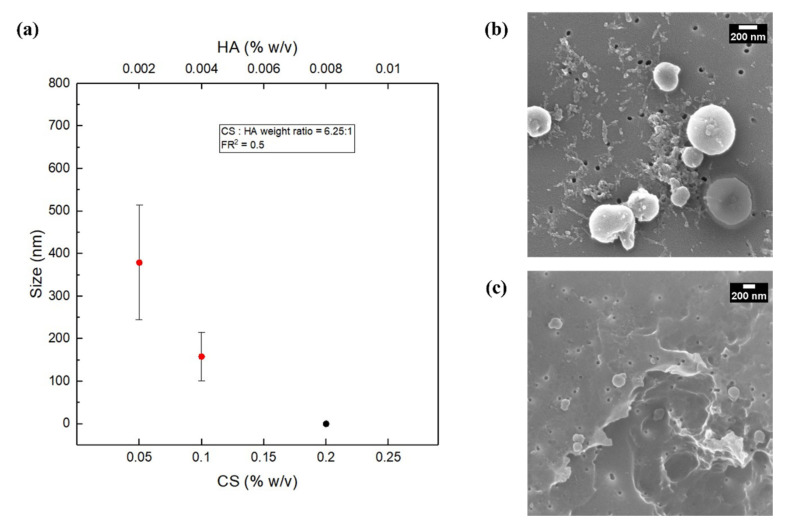
NPs’ size and morphology at constant polymer ratio reproducing batch conditions: (**a**) Plot showing size vs. CS and HA concentrations at CS: HA weight ratio equal to 6.25 at low flow rate regime (MFR = 0.3 μL/min and SFR = 0.6 μL/min). The black point indicates that only macroaggregates and unreacted materials are obtained, therefore the size was not measurable (see Figure S3 of the Supplementary Materials); (**b**) CS = 0.05% *w*/*v*; (**c**) CS = 0.1% *w*/*v*.

Most experiments have been carried out in two different operating flow rate regimes (Figure 1b,c). The operating regimes were defined in two steps. Firstly, we identified a threshold after which we visually observed, through an optical microscope, a change in focusing width together with a shift in the position of the relative focusing. These observations allowed us to experimentally choose two conditions: (1) a low flow rate regime, i.e., middle channel flow rate < 1 μL/min, flow focusing width *w* below 15 μm and a mixing time $\tau_{mix}$ below 30 ms (Figure 1b); (2) a high flow rate regime, i.e., middle channel flow rate ≥ 1 μL/min, flow focusing width *w* above 15 μm and a mixing time $\tau_{mix}$ above 30 ms (Figure 1c). In both cases, we worked in a mixing time range in the order of tens of milliseconds, which is typically used for polysaccharide nanoparticle fabrication [74]. The experimentally set threshold allows us to investigate two processes characterized by longer and shorter mixing times, respectively, higher and lower than the aggregation time reported for CS-based nanoparticles [75,76].

### 3.3. Rational of the Experimental Campaign on Ionotropic Gelation in Microfluidics

Before approaching the experimental campaign in microfluidics, a detailed analysis of the literature related to the batch processes implementing ionotropic gelation has been performed to identify the optimal thermodynamic conditions to implement in microfluidics.

Consequently, we found that a standard parameter used in ionotropic gelation batch processes is the polymer ratio, usually kept constant at 6.25 (CS:HA = 6.25:1 weight ratio), as described by Callewaert et al. [23]. The translation of this condition to microfluidics has been obtained by controlling the flow rates and, therefore, the flow rate ratio (details on the calculation of the polymer weight ratio within the microchannels with respect to the flow rate are reported in Equation (S1) and Figure S1 of the Supplementary Materials).

In particular, we investigated how the gap from the saturation concentration of the compounds affects both the nanoprecipitation and the ionotropic gelation, influencing the diffusion and electrostatic coupling of the polymers.

Among the tested conditions and parameters (listed in Table S1 of the Supplementary Materials), we focused our work on those that experimentally allowed reproducibility of the results, stability of the hydrodynamic flow focusing and high throughput. In particular, an FR^2^ of 0.5 has been chosen as the most reliable value to carry out the process. In detail, by keeping the FR^2^ constant, the experiments in the low flow rate regime have been conducted at the middle channel flow rate of 0.3 μL/min and side channels of 0.6 μL/min. On the other hand, experiments in the high flow rate regime have been performed in the middle channels at a flow rate of 3 μL/min and the side channels at 6 μL/min. The effect of polymer concentrations (ranging from 0.05 to 0.2% *w*/*v* for CS and from 0.002 to 0.008% *w*/*v* for HA) and polymer ratios (CS:HA ranging from1.56 to 6.25) at set FR^2^ conditions have been investigated as are discussed in the following at high and low flow rate regimes. Representative results obtained using other FR^2^ conditions are displayed in Figure S2 of the Supplementary Materials.

### 3.4. Effect of the Concentration of the Polymers at FR^2^ = 0.5 and Constant Polymer Ratio of 6.25

Firstly, by keeping the FR^2^ constant at 0.5, the effect of increasing CS and HA concentrations at a constant weight ratio (CS:HA = 6.25:1) have been explored at low and high flow rate regimes. As stated above, this primary ratio was selected because widespread used in the literature. It was scaled to be adapted at the microfluidic conditions.

Z-Average Size obtained by DLS and SEM images, reported in Figure 2, reveal the presence of coacervates, whose size and polydispersity decrease with the increasing CS concentration, which is an unexpected phenomenon since the increase in concentration usually brings an increase in the particles size. Indeed, a higher polymer concentration improves the viscosity of the organic phase, which eventually reduces its diffusion rate towards the aqueous phase, subsequently resulting in larger nanoparticles [77].

An explanation can be found in the enhancement of the stability of the hydrodynamic flow focusing. Indeed, even a slight increment of the concentrations of the polymer induces the increase in the viscosity, the reduction in the fluctuation of the focusing and better control of the nanoprecipitation, promoting nucleation to the detriment of growth [70]. This interpretation is confirmed by the results at the highest CS concentrations, equal to 0.2% *w*/*v*. The latter condition, indeed, produces a stable focusing but causes a massive precipitation and promotes the formation of big aggregates instead of NPs [67,78]. In the high flow rate regime, a similar behavior was observed.

### 3.5. Effect of the Polymer Ratio at FR^2^ = 0.5

Effect of polymer concentrations (ranging from 0.05 to 0.2% *w*/*v* for CS and from 0.002 to 0.008% *w*/*v* for HA) and polymer ratios (CS:HA ranging from1.56 to 6.25) at set FR^2^ conditions of high and low flow rate regimes have been investigated as discussed in the following.

**Figure 3:**
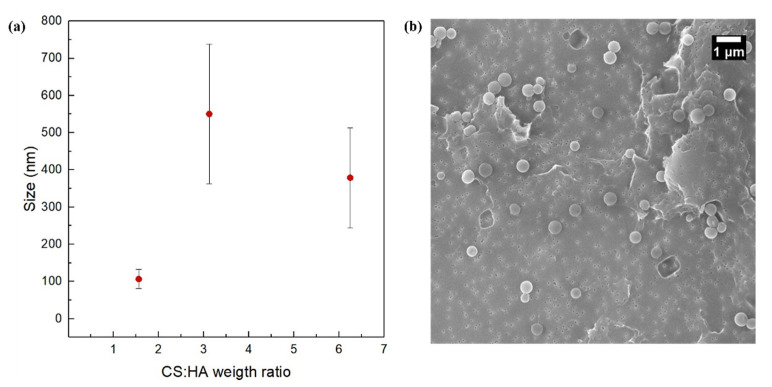
NPs’ size and morphology at different polymer ratios at low flow rate regime and FR^2^ = 0.5: (**a**) Plot of NP size vs. CS:HA weight ratio; (**b**) representative SEM image at CS: HA weight ratio equal to 3.12.

**Figure 4:**
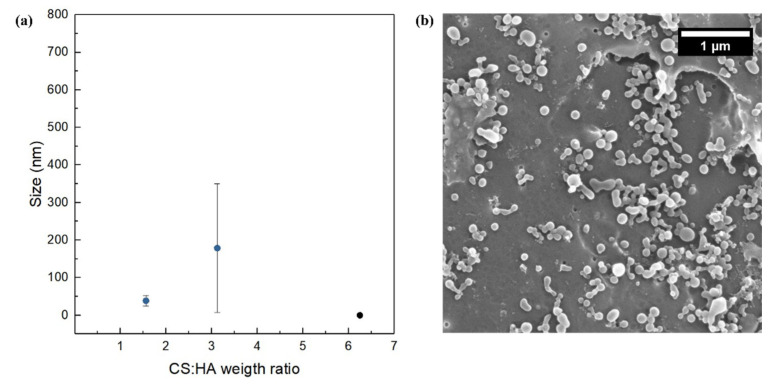
NPs’ size and morphology at different polymer ratios at high flow rate regime and FR^2^ = 0.5: (**a**) Plot of NP size vs. CS:HA weight ratio; (**b**) representative SEM image at CS: HA weight ratio equal to 1.56. The black point indicates that, at high flow rate regime and CS:HA = 6.25, macroaggregates and unreacted materials are obtained; therefore, the size was not measurable (see Figure S4 of the Supplementary Materials).

In details, polymer ratios CS:HA equal to 1.56 (CS = 0.0125% *w*/*v*, HA = 0.002% *w*/*v*; CS = 0.05% *w*/*v*, HA = 0.008% *w*/*v*) and CS:HA equal to 3.12 (CS = 0.1% *w*/*v*, HA = 0.008% *w*/*v*; CS = 0.025% *w*/*v*, HA = 0.002% *w*/*v*) were analyzed. Results show that, regardless of the flow rate regimes, size grows when polymer ratio increases (Figure 3 and Figure 4).

The explanation can be found in the role played by the nucleation phenomenon. Indeed, it has been already reported that the higher the concentrations of the polymers, the higher the size of starting nuclei and the more the growth phenomena are favored [55]. Moreover, it is worth noticing how the size of the NPs increases by shifting from the high flow rate regime to low flow rate regime. In this case, we visually observe that, in the low flow rate regime, the hydrodynamic flow focusing starts closer to the chip junction, promoting physical aggregation. It means that polymer availability lasts longer at the channel crossing, promoting aggregation more than nucleation and so leading to a higher mean size of the NPs. A similar effect has also been reported previously by Nemati et al. [79] for the use of microfluidics to tune the size and shape of chitosan NPs adsorbing Hg from aqueous solutions for environmental applications. This effect can also be compared with results obtained by other authors in different contexts and proving the fine tunability of the process parameters in microfluidics [78,80,81].

### 3.6. Interpretation of the Operating Regimes and Obtained Morphologies

**Figure 5:**
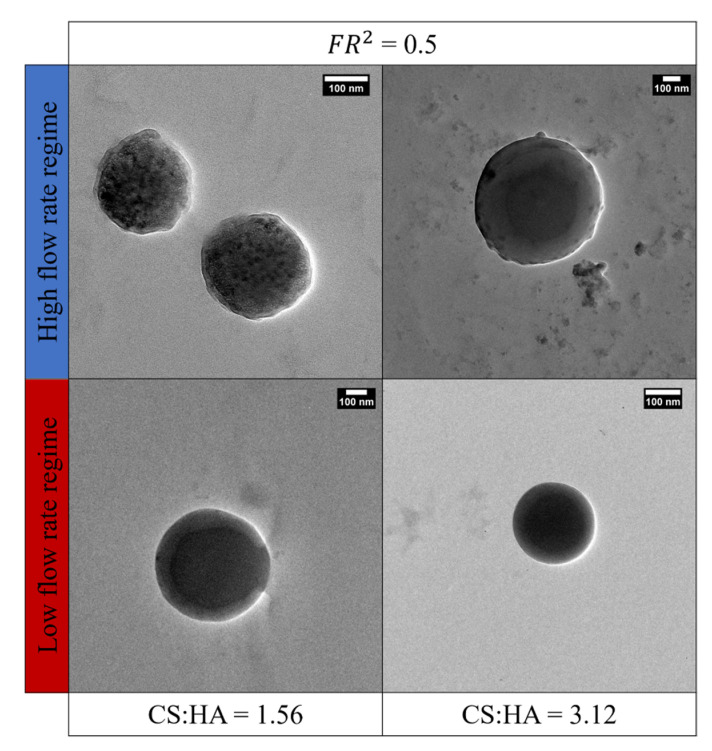
NPs’ morphologies obtained by TEM characterization for different CS:HA weight ratios and flow rate regimes and at constant FR^2^. Sizes range from: upper left 38.57 ± 14.31 nm; upper right 178.63 ± 171.47 nm; lower left 471.86 ± 67.28 nm; lower right 150.33 ± 112.01 nm (size distribution histograms are displayed in Figure S5 of the Supplementary Materials).

A comparison between the morphologies obtained at FR^2^ = 0.5 and two CS:HA ratios at high and low flow rate regimes is shown in Figure 5.

In Figure 5, it is possible to observe that the high flow rate regime promotes the transition to different morphologies such as coprecipitate and core–shell NPs thanks to parameter modulation, whereas tuning the conditions within the low flow rate regime leads to NPs with a mainly core–shell morphological structure. In the low flow rate regime, we also showed that it is possible to change the overall size of the core–shell nanostructure as well as the relative dimensions between the core and shell area. Other morphologies have also been investigated by varying the TPP concentration (Figure S6 of the Supplementary Materials), and two visual plots with additional TEM images have been included in the Supplementary Materials (Figures S7 and S8).

These results highlight microfluidics’ ability to tune the parameters to obtain a range of architectures attractive from both industrial and research perspectives in the nanomedicine field [81]. This ability is determined by the competition between the fluidodynamic forces and the thermodynamics of ionotropic gelation.

### 3.7. Understanding the Role of Fluododynamic Regimes in Ionotropic Gelation Implemented in Microfluidics

**Figure 6:**
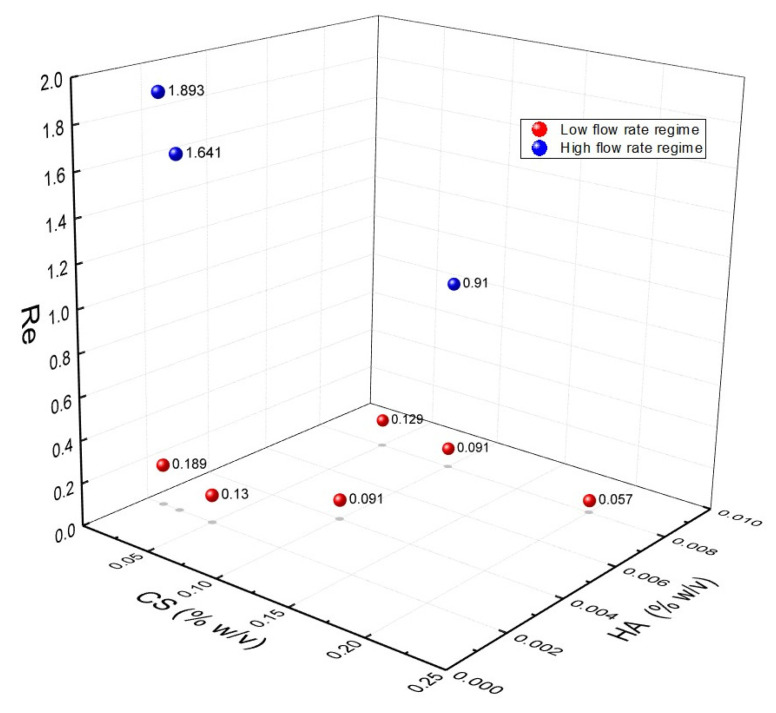
Characterization of the fluidodynamic regime through Reynolds numbers at different polymer concentrations at low (0.6–0.3–0.6 μL/min) and high (6–3–6 μL/min) flow rate regimes.

The effect of the flow rate regimes has been interpreted by analyzing the Reynolds number (Re) [82]. Taking into account the defined 1 μL/min threshold, Re values at FR^2^ = 0.5 have been calculated approximately considering two reproducible conditions: 0.6–0.3–0.6 μL/min and 6–3–6 μL/min (side channel–middle channel–side channel). Figure 6 shows the Reynold numbers at different CS and HA concentrations and distinguishing between high and low flow rate regimes (additional graphs on the relationships between the Reynold number and the CS concentration are reported in Figures S9 and S10 of the Supplementary Materials).

Results show that laminar flow within the device is guaranteed. However, a threshold has been observed, which identifies the high and low flow rate regimes at Re > 1 and Re ≤ 1, respectively. In the case of the high flow rate regime, phenomena are driven by the fluid velocity and, therefore, the mixing processes are faster than coacervation also producing coprecipitated morphologies, while, in the low flow rate regime, the properties of the materials are predominant and, therefore, the viscous forces drive the coacervation phenomena. This explanation is also confirmed by the borderline condition reached at Re = 0.91. In this condition, which corresponds to CS:HA = 3.12, despite being in the high flow rate regime, an equilibrium between the inertial and viscous forces is attained and core–shell morphologies are produced, as already reported in Figure 5.

### 3.8. Encapsulation Efficiency, Cytotoxicity and Multimodal Properties of the Hydrogel Nanostructures

#### 3.8.1. In Vitro MRI

Gadolinium has been introduced in the process starting from the best conditions identified for NP formation (FR^2^ = 0.5, HA = 0.008% *w*/*v*, CS = 0.1% *w*/*v*, TPP = 0.012% *w*/*v* and CS: HA = 3.12). The reproducibility of the trial, the high throughput and the observed core–shell morphology have been the selected parameters that justified this choice. Gadolinium has been added to the polycationic solutions with a concentration empirically set equal to that of CS (Gd = 0.1% *w*/*v*).

After dialysis, the size of the NPs slightly increases due to the presence of Gd, which attracts a high number of water molecules into the polymer matrix, leading to larger NPs. For this reason, the concentration of the Gd-DTPA within the NPs was measured by ICP-MS and then compared to the initial concentration used in the process to estimate the encapsulation efficiency (EE) according to the following formula [83]:

$$\mathrm{EE}\;=\;(\frac{C_{en}}{C_{i}})\;\times \;100$$

where $C_{en}$ is the Gd-DTPA concentration measured by ICP-MS and $C_{i}$ is the theoretical Gd-DTPA concentration used in the process.

ICP-MS values proved that Gd-DTPA is entrapped within the polymeric matrix, giving an estimated EE equal to 11.95% (Table S3 of the Supplementary Materials).

**Figure 7:**
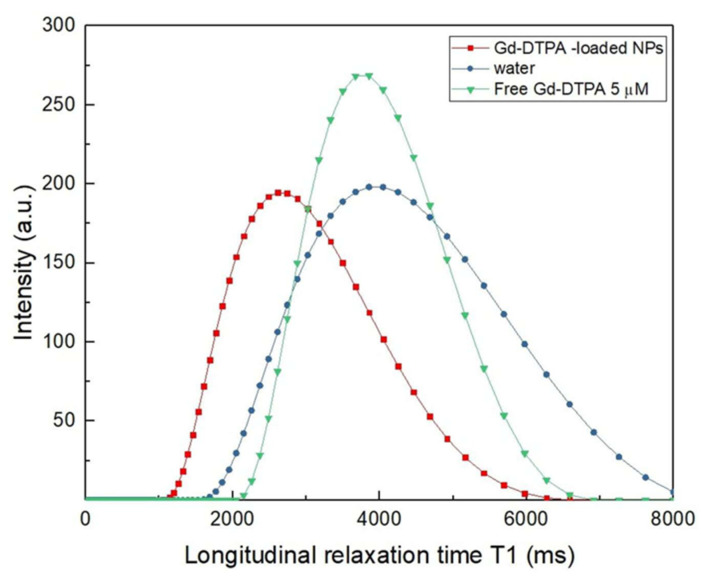
Relaxation time curves for Gd-DTPA-loaded NPs in water compared to free water and to the corresponding free Gd-DTPA in water.

Additionally, the longitudinal relaxation time, $T_{1}$, of the Gd-DTPA loaded NPs was measured using a benchtop relaxometer and compared with the $T_{1}$ of both water and free Gd-DTPA in water (Figure 7).

By comparing the $T_{1}$ distribution of the Gd-DTPA loaded NPs with the $T_{1}$ distribution of the corresponding free Gd-DTPA in water as measured by ICP-MS (Gd-DTPA = 5 µM), it results in the loaded NPs having a 3.8-fold higher relaxivity, corresponding to a 12.3% enhancement in the longitudinal relaxation rate (Table S4 of the Supplementary Materials). These values, interpreted in the framework of the Hydrodenticity concept, are the results of the water-mediated interaction between the polymer and metal chelate. In detail, the hydrophilic behavior of both polymers allows the accumulation of a large amount of water inside the structure, increasing interactions between water molecules and the metal chelate due to the presence of the polymeric matrix that affects water molecules dynamics. This improved hydration degree of Gd-DTPA leads to a relaxivity boosting of the CA. An enhancement of the $T_{1}$ value translates into an improvement of the intensity of the MRI signal and so a better contrast between distinct tissues. Further experiments on gadolinium encapsulation are reported in the Supplementary Materials (Table S2). Based on these results, future works will aim at conducting additional trials to quantify the relationship between the structural (size and morphology of the nanoparticles) and the functional (relaxometric parameters) properties of the designed nanovectors.

#### 3.8.2. In Vitro Optical Imaging

Atto-488 fluorophore has been added to the polyanionic solution (35 μg/mL) in order to demonstrate that NPs are able to encapsulate, in a one-step process, two imaging agents. The Atto-488 encapsulated amount has been evaluated through spectrofluorimetric measurements, which show an estimated concentration of 7 pmol/mL (EE = 16.1%). See also Figure S11 of the Supplementary Materials as a reference for the spectrofluorimeter measurement. Moreover, preliminary viability of U87 cells exposed to HA–CS nanoparticles, obtained at the process conditions of FR^2^ = 0.5, HA = 0.008% *w*/*v*, CS = 0.1% *w*/*v*, TPP = 0.012% *w*/*v* and CS: HA = 3.12, are displayed in Figure S12. Results showed no significant cytotoxicity at different time point up to 24 h of incubation for a nanoparticle concentration higher than 20 µgr/mL

## 4. Discussion

The ability to design and engineer specific nanostructures allows us to reach and treat disease with cellular and molecular precision. Through the optimal design of the nanoparticles, it is possible to improve the nano–bio interactions and to overcome limits of the tissue specificity and stability of the active compounds, enhancing the diagnostic imaging window, reducing the administration dosage and, at the same time, increasing the theranostic performances.

Microfluidics represents a promising tool to finely synthesize libraries of nanoparticles in a controlled, reproducible, high-throughput manner, thus accelerating their translation to a relevant clinical environment. In this paper, we adopted a microfluidic HFF approach to form NPs by ionotropic gelation of CS and TPP, followed by complex coacervation between CS and HA.

We demonstrate that, by varying process parameters (flow rates, polymer concentrations, polymer weight ratio and crosslinking degree), it is possible to modulate the size of NPs and their morphologies and structures. During the experimental campaign, a wide range of these process parameters has been explored to investigate their influence on the ionotropic gelation reaction and on the resulting nanoarchitectures.

Compared to batch processing, the implemented microfluidic process requires lower polymer concentrations, in agreement with other studies comparing batch protocols with microfluidic processes [84,85,86,87]. In addition, the proposed strategy allows optimizing the interaction among the chosen compounds. Indeed, very high polymer concentrations could be a limiting factor in the formation of hybrid NPs within microchannels because the accumulation of materials at the chip junction results in a reduction in the process controllability and in the consequent increase in polydispersity, or even in the formation of aggregates rather than NPs.

In our work, both the polymer concentrations (CS and HA), as shown in Figure 2, and their weight ratio (CS:HA), as shown in Figure 3 and Figure 4, can affect the output of the process. In fact, while at a fixed polymer ratio the increase in concentration brings a reduction in size and polydispersity (Figure 2), on the other hand, by increasing the polymer ratio, the growth phenomenon goes in the opposite direction, leading to bigger and polydisperse nanostructures regardless of the chosen flow rate regime (Figure 3 and Figure 4).

The crosslinking degree, combined with variations in flow rates, proved to be another controllable parameter to modulate size and morphology of the NPs, as shown in Figure S6. This is due to their influence on the binding process between anionic and cationic blocks. High TPP concentration, i.e., higher availability of the crosslinker, does not always lead to the formation of more stable and smaller NPs if it is not balanced by appropriate flow rates, in accordance with Whiteley et al. [84].

As a result of these considerations, morphological and structural features of the NPs are the outcome of the modulation of different factors involved in the microfluidic ionotropic gelation process, as shown in Figure 5, which demonstrates how the chosen process parameters enable an ease shift in the architecture changing from coprecipitates to different core–shell structures. Such a design strategy, pushed to the molecular level, not only leads to a fine control over the nanoarchitecture but, above all, it is also crucial in determining the functional properties of the nanocarrier, making it relevant not only in the field of precision medicine but also in other areas of medicine and biology [88,89,90,91,92]. Indeed, a considerable advantage of the proposed approach consists of the opportunity to obtain a relaxivity boost of the encapsulated CAs for MRI (Figure 7) by changing the structural parameter of the hydrogel matrix and exploiting the Hydrodenticity concept, as underlined by Russo et al. [31]. The attainment of the Hydrodenticity within the nanostructure influences the characteristic correlation times of the Solomon–Bloembergen–Morgan theory [93,94] and, consequently, it enables an increase in the relaxivity of the Gd-DTPA.

The MRI signal boost, here shown as preliminary results in Figure 7, is interpreted in terms of Hydrodenticity, induced by the modulation of mainly the intra and inter crosslinking between the polymers and their different hydrophilicity. In our process, the hydration mechanism of the Gd-DTPA can be tuned by changing, through microfluidics, the sub-architecture of the nanostructures made of chemical interconnections between the two polymers. Indeed, the possibility to move from coprecipitate to core–shell and to control inner and outer diameters of a core–shell NP, as well as the opportunity to change the hydrophilicity of the NPs by increasing the amount of HA in the coprecipitates nanostructures, allows controlling metal-chelate hydration and tuning its relaxometric properties.

As a result, this approach introduces a powerful tool to control and take advantage of the complex structure–function relationship that characterizes the nanoarchitectures.

## 5. Conclusions

In this work, we proved that microfluidic parameters can be tuned to control not only the nanoparticles’ size, but also their morphologies and physicochemical properties, potentially dictating their biological fate.

Here, starting with the study of batch process conditions, a one-step hydrodynamic flow-focusing process to produce CS–HA NPs by ionotropic gelation is implemented in a custom-designed microfluidic platform to obtain tailored structures and morphologies by tuning the process parameters. The control over the gelation reaction, occurring in the microfluidic chip, is achieved by changing the flow rates of the inlets, the volumetric flow rate ratio and the ratio of the different compounds adopted (polymers and crosslinker), producing a variety of nanostructures with different morphologies.

The advantages of the microfluidic flow focusing approach in the design of HA–CS NPs lie, on the one hand, in the possibility to overcome the drawbacks of batch processes (time-consuming, multiple-step processes, higher consumption and waste of unreacted material, poor control overreaction and overall process performance), offering, on the other hand, the possibility to customize the nanovectors by tuning the process parameters. However, the proper microfluidic translation of such a complex process, i.e., the ionotropic gelation, has to consider the extremely crucial role of fluidodynamics. Indeed, diffusion and thermodynamic phenomena in the microfluidic chip, occurring together with ionic and electrostatic interactions, produce variations in the process up to the molecular level and have a leading role in the formation of the nano-architecture, which mostly determines the properties of the nanocarrier and its eligibility for the desired application.

Preliminary data on the simultaneous encapsulation of both a gadolinium-based CA for MRI and a dye (Atto-488) for Optical Imaging are also shown, suggesting the potential use of these hybrid nanocarriers in the multimodal imaging field.

These findings are addressing the optimal design in the precision nanomedicine field and theranostics. These results could be useful to show up the power of microfluidics in building up a library of nanovectors by fine tuning fluidodynamic and thermodynamic parameters. 

## Data Availability

The data presented in this study are available in the Supplementary Materials.

## Supplementary Materials

The following are available online at https://www.mdpi.com/article/10.3390/biomedicines9111551/s1, Figure S1: Polymer weight ratio calculated within the microchannel. The dashed square represents the control volume with polymer mass rates flowing in and out per unit time, Table S1: Parameters tested during the experimental campaign. (HA = Hyaluronic Acid; CS = Chitosan; TPP = Sodium Tripolyphosphate), Figure S2: Representative SEM images of flow rate ratio conditions tested, Table S2: Parameters tested during the experimental campaign after the addition of Gd-DTPA, Figure S3: Representative SEM image of CS 0.2% *w/v* at CS:HA=6.25 and FR^2^ = 0.5, Table S3: Calculation of encapsulation efficiency starting by the measurements taken from the ICP-MS, Figure S4: Representative SEM images of different CS:HA ratios at FR^2^ = 0.5, Table S4: Calculation of relaxation enhancement starting by the measurements taken from the the benchtop relaxometer, Figure S5: Representative histograms and distribution curves of NPs at FR^2^ = 0.5 and CS:HA ratio equal to 1.56 or 3.12 at the two operational regimes, Figure S6: NPs size as a function of FR^2^ (0.05, 0.2 and 0.4) at two TPP concentration (0.012 or 0.003% *w/v*) and SEM images of NPs at two TPP concentrations and two different flow rates, Figure S7: Visual plot displaying morphology of NPs at low flow rate regime as a function of FR^2^ and CS:HA ratio, Figure S8: Visual plot displaying morphology of NPs at high flow rate regime as a function of FR^2^ and CS:HA ratio, Figure S9: Reynolds number as a function of the chitosan concentration at high and low flow rate regime, Figure S10: Reynolds number as a function of the total flow rate at different chitosan concentrations, Figure S11: Spectrofluorometer calibration curve for Atto-488 in the range 0-200 pmol/mL, Figure S12: In vitro cytotoxicity of CS-HA NPs in contact with U87 MG cells at two different time points.
