# Supplementary material for: Tuning of Hydrogel Architectures by Ionotropic Gelation in Microfluidics: Beyond Batch Processing to Multimodal Diagnostics
Source: Biomedicines. 2021 Oct 27;9(11):1551. doi: 10.3390/biomedicines9111551 (PMC8614968; doi:10.3390/biomedicines9111551)
Supplement: Supplementary file 1 [file biomedicines-09-01551-s001.zip › biomedicines-1387847-supplementary.pdf]

# SUPPLEMENTARY MATERIAL

# Tuning of hydrogel architectures by ionotropic gelation in Microfluidics: beyond batch processing to multimodal diagnostics

**Alessio Smeraldo [1,2], Alfonso Maria Ponsiglione [1] ,Paolo Antonio Netti [1,2,3] and Enza Torino [1,2,3,*]**

[1]*Department of Chemical, Materials and Production Engineering, University of Naples Federico II, Piazzale Tecchio 80, 80125 Naples, Italy*
[2]*Center for Advanced Biomaterials for Health Care, CABHC, Istituto Italiano di Tecnologia, IIT@CRIB, Largo Barsanti e Matteucci 53, 80125 Naples, Italy*
[3]*Interdisciplinary Research Center on Biomaterials, CRIB, Piazzale Tecchio 80, 80125 Naples, Italy*

**Variation of the process parameters.**

During the experimantal campaign, the main process parameters that have been tested are reported in the following Table S1.

**Table S1.** Parameters tested during the experimental campaign. (HA = Hyaluronic Acid; CS = Chitosan; TPP = Sodium Tripolyphosphate)

| Test # | HA (%w/v) | TPP (%w/v) | CS (%w/v) | Flow rate middle channel (µL/min) | Flow rate side channel (µL/min) | Flow Rate Ratio (FR[2]) | CS:HA (g/g) |
|---|---|---|---|---|---|---|---|
| 1 | 0.008 | 0.012 | 0.025 | 20 | 100 | 0.2 | 0.312 |
| 2 | 0.008 | 0.012 | 0.025 | 20 | 100 | 0.2 | 0.312 |
| 3 | 0.008 | 0.012 | 0.025 | 0.75 | 5 | 0.15 | 0.234 |
| 4 | 0.004 | 0.006 | 0.0125 | 0.75 | 5 | 0.15 | 0.234 |
| 5 | 0.004 | 0.006 | 0.0125 | 20 | 100 | 0.2 | 0.312 |
| 6 | 0.002 | 0.003 | 0.00625 | 20 | 100 | 0.2 | 0.312 |
| 7 | 0.002 | 0.003 | 0.00625 | 0.75 | 5 | 0.15 | 0.234 |
| 8 | 0.002 | 0.003 | 0.00625 | 20 | 100 | 0.2 | 0.312 |
| 9 | 0.002 | 0.003 | 0.00625 | 20 | 100 | 0.2 | 0.312 |
| 10 | 0.002 | 0.003 | 0.00625 | 20 | 100 | 0.2 | 0.312 |
| 11 | 0.002 | 0.003 | 0.00625 | 0.75 | 5 | 0.15 | 0.234 |
| 12 | 0.002 | 0.003 | 0.00625 | 20 | 100 | 0.2 | 0.312 |
| 13 | 0.002 | 0.003 | 0.00625 | 0.75 | 5 | 0.15 | 0.234 |
| 14 | 0.002 | 0.003 | 0.00625 | 3(CS) - 9(HA) * | 90 | - | - |
| 15 | 0.002 | 0.003 | 0.00625 | 20 | 100 | 0.2 | 0.3125 |

| | | | | | | | |
|---|---|---|---|---|---|---|---|
| 16 | 0.002 | 0.003 | 0.00625 | 3(CS) - 9(HA) * | 90 | - | - |
| 17 | 0.002 | 0.003 | 0.00625 | 0.3 | 4 | 0.075 | 0.117 |
| 18 | 0.002 | 0.003 | 0.00625 | 0.2 | 4 | 0.05 | 0.0781 |
| 19 | 0.002 | 0 | 0.00625 | 20 | 100 | 0.2 | 0.312 |
| 20 | 0.004 | 0.006 | 0.0125 | 0.15 | 2.5 | 0.06 | 0.0937 |
| 21 | 0.002 | 0.003 | 0.00625 | 0.2 | 4 | 0.05 | 0.0781 |
| 22 | 0.002 | 0.003 | 0.00625 | 0.2 | 4 | 0.05 | 0.0781 |
| 23 | 0.002 | 0.003 | 0.00625 | 0.2 | 4 | 0.05 | 0.0781 |
| 24 | 0.002 | 0.003 | 0.00625 | 0.2 | 4 | 0.05 | 0.0781 |
| 25 | 0.002 | 0.003 | 0.00625 | 0.2 | 1 | 0.2 | 0.312 |
| 26 | 0.002 | 0.003 | 0.00625 | 0.2 | 1 | 0.2 | 0.312 |
| 27 | 0.002 | 0.003 | 0.00625 | 0.2 | 1 | 0.2 | 0.312 |
| 28 | 0.002 | 0.003 | 0.00625 | 0.2 | 0.5 | 0.4 | 0.625 |
| 29 | 0.002 | 0.012 | 0.00625 | 0.2 | 1 | 0.2 | 0.312 |
| 30 | 0.002 | 0.012 | 0.00625 | 10 | 25 | 0.4 | 0.625 |
| 31 | 0.002 | 0.012 | 0.00625 | 0.2 | 4 | 0.05 | 0.0781 |
| 32 | 0.002 | 0.012 | 0.00625 | 0.2 | 0.5 | 0.4 | 0.625 |
| 33 | 0.002 | 0.003 | 0.00625 | 10 | 25 | 0.4 | 0.625 |
| 34 | 0.002 | 0.012 | 0.00625 | 0.2 | 1 | 0.2 | 0.312 |
| 35 | 0.002 | 0.003 | 0.00625 | 0.2 | 0.5 | 0.4 | 0.625 |
| 36 | 0.002 | 0.003 | 0.00625 | 0.2 | 1 | 0.2 | 0.312 |
| 37 | 0.002 | 0.012 | 0.00625 | 10 | 25 | 0.4 | 0.625 |
| 38 | 0.002 | 0.012 | 0.00625 | 2 | 5 | 0.4 | 0.625 |
| 39 | 0.008 | 0.012 | 0.2 | 0.3 | 0.6 | 0.5 | 6.25 |
| 40 | 0.004 | 0.006 | 0.1 | 0.3 | 0.6 | 0.5 | 6.25 |
| 41 | 0.002 | 0.003 | 0.05 | 0.3 | 0.6 | 0.5 | 6.25 |
| 42 | 0.008 | 0.012 | 0.05 | 0.3 | 0.6 | 0.5 | 1.56 |
| 43 | 0.008 | 0.012 | 0.1 | 0.3 | 0.6 | 0.5 | 3.12 |
| 44 | 0.002 | 0.003 | 0.0125 | 0.3 | 0.6 | 0.5 | 1.56 |
| 45 | 0.002 | 0.003 | 0.0125 | 3 | 6 | 0.5 | 1.56 |
| 46 | 0.008 | 0.012 | 0.1 | 3 | 6 | 0.5 | 3.12 |
| 47 | 0.008 | 0.012 | 0.1 | 3 | 6 | 0.5 | 3.12 |
| 48 | 0.008 | 0.012 | 0.1 | 0.3 | 0.6 | 0.5 | 3.12 |
| 49 | 0.002 | 0.003 | 0.025 | 3 | 6 | 0.5 | 3.12 |

* attempts of CS-HA pre-mixing: both CS and HA flow in the middle channel while pure water flows in side channels.

Concentration of the three compounds used (HA = Hyaluronic Acid; CS = Chitosan; TPP = Sodium Tripolyphosphate) in the process are reported in the first three columns while the process parameters (Middle and Side Flow Rates and their ratio) are shown in the following three. In the last column, the CS:HA weight ratio at the junction is reported, considering that HA concentration is doubled due to its presence in both side channels (a more detailed explanation of the ratio for the calculation of the polymer ratio at the channel junction is given in the following paragraph).

**Rationale for the calculation of the polymer weight ratio within microchannels.**

In order to calculate the polymer weight ratio obtained within the channel of the microfluidic device, the following equation (Eq. S1) has been used:

$$Polymer\ weight\ ratio\ within\ microchannels = \frac{\dot{m}_{CS}}{2 \times \dot{m}_{HA}} \quad with \quad \begin{cases} \dot{m}_{CS} = [CS] \times FR_{CS} \\ \dot{m}_{HA} = [HA] \times FR_{HA} \end{cases} \qquad \text{(Eq. S1)}$$

where [CS] and [HA] are the concentrations (expressed in μg/μL) while $FR_{CS}$ and $FR_{HA}$ are the flow rates (expressed in μl/min) used for Chitosan and Hyaluronic Acid respectively.

The following Figure S1 is a schematic representation of the calculation of the polymer weight ratio obtained right after the fluid mixing at the chip junction:

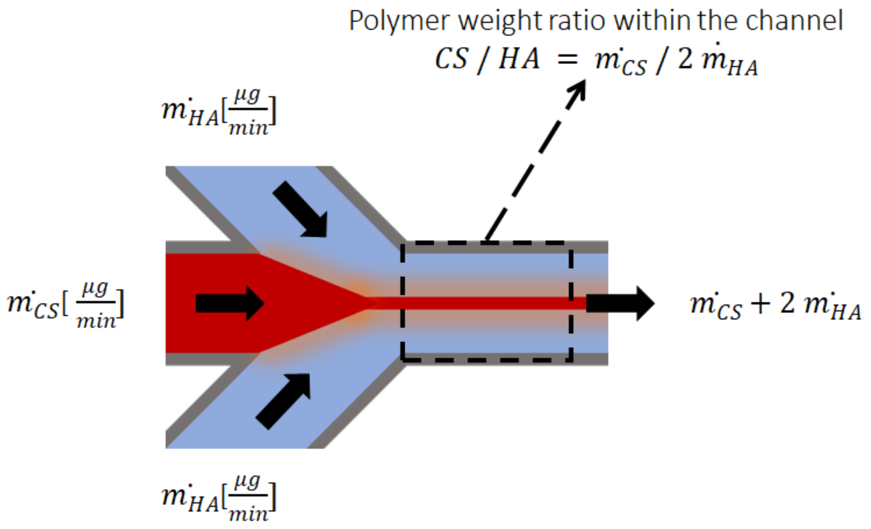


**Figure S1.** Polymer weight ratio calculated within the microchannel. The dashed square represents the control volume with polymer mass rates flowing in and out per unit time.

The aim of this rationale is reproducing batch conditions of the ionotropic gelation process within a microfluidic device using a hydrodynamic flow focusing approach. The control volume in Figure S1 simulates the batch where the polymer solutions are mixed and the microfluidic flow focusing is carried out keeping proportions and ratios used for batch processes.

**Identification of optimal flow rate ratio condition.**

The best conditions of the flow rate ratio are seleceted as those allowing reproducibility of the results, high nanoparticles throughput and stability of the flow focusing. In the following Figure S2, we show representative images of the different conditions tested.

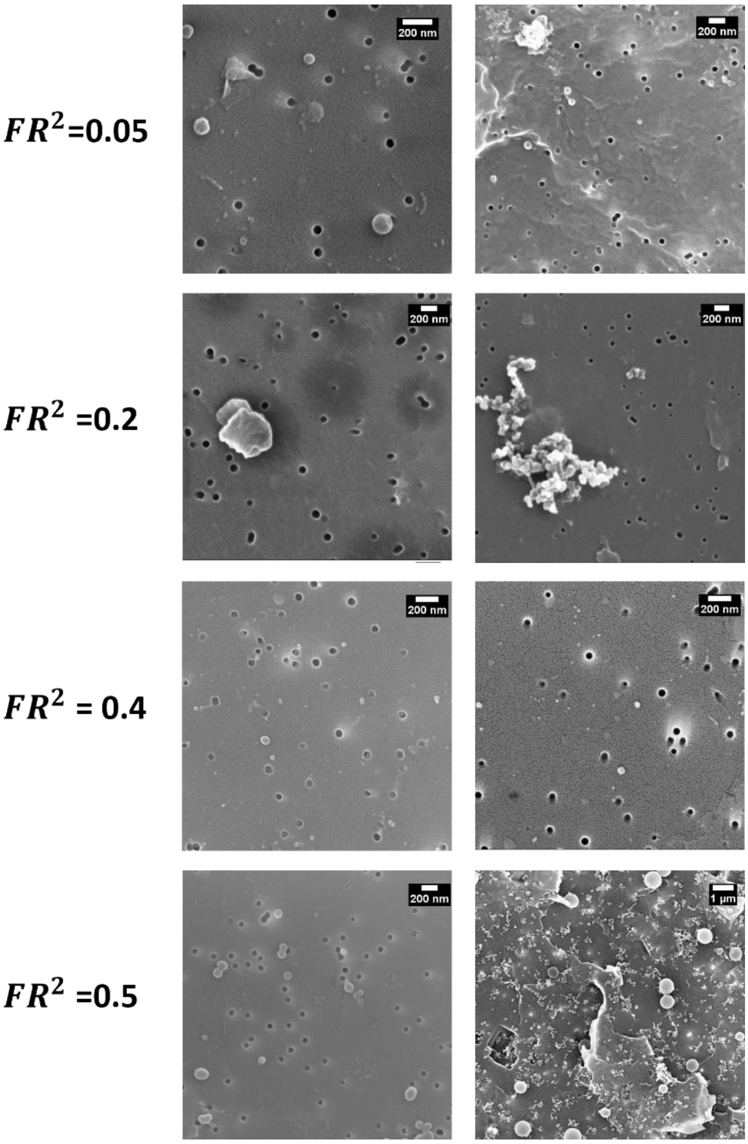


**Figure S2.** Representative SEM images of flow rate ratio conditions tested.

As from Figure S2, $FR^2$ = 0.5 is selected as the optimal condition leading to numerous and reproducible nanoparticles.

**Effect of the concentration of the polymers at** FR$^2$ **= 0.5 and constant polymer ratio.**

Experiment carried out at CS=0.2 %w/v ,CS:HA=6.25 and FR$^2$=0.5 is showed in the following Figure S3.

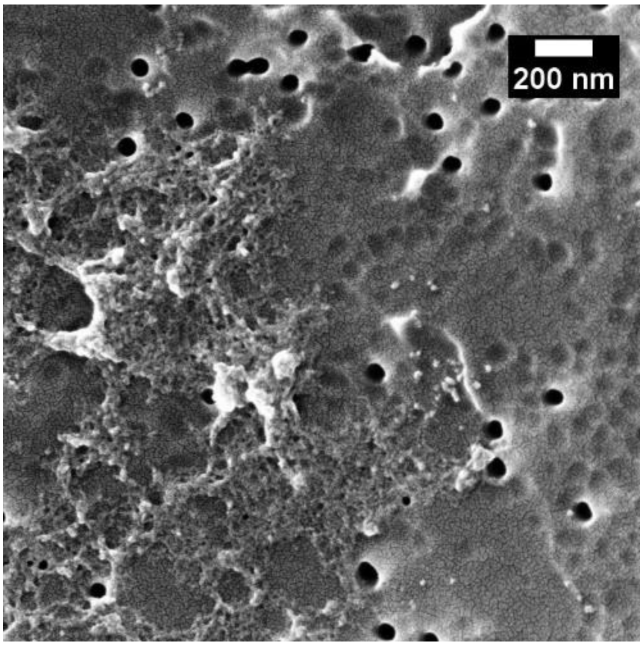


**Figure S3.** Representative SEM image of CS 0.2 %w/v at CS:HA=6.25 and FR$^2$ = 0.5.

Macroaggregates and unreacted materials are obtained in this case instead of nanoparticles, therefore the size was not measurable.

**Effect of the polymer ratio at** $FR^2$ **= 0.5.**

The following figure displays SEM images related to different CS:HA ratios at *High* and *Low flow rate regimes* at costant $FR^2 = 0.5$.

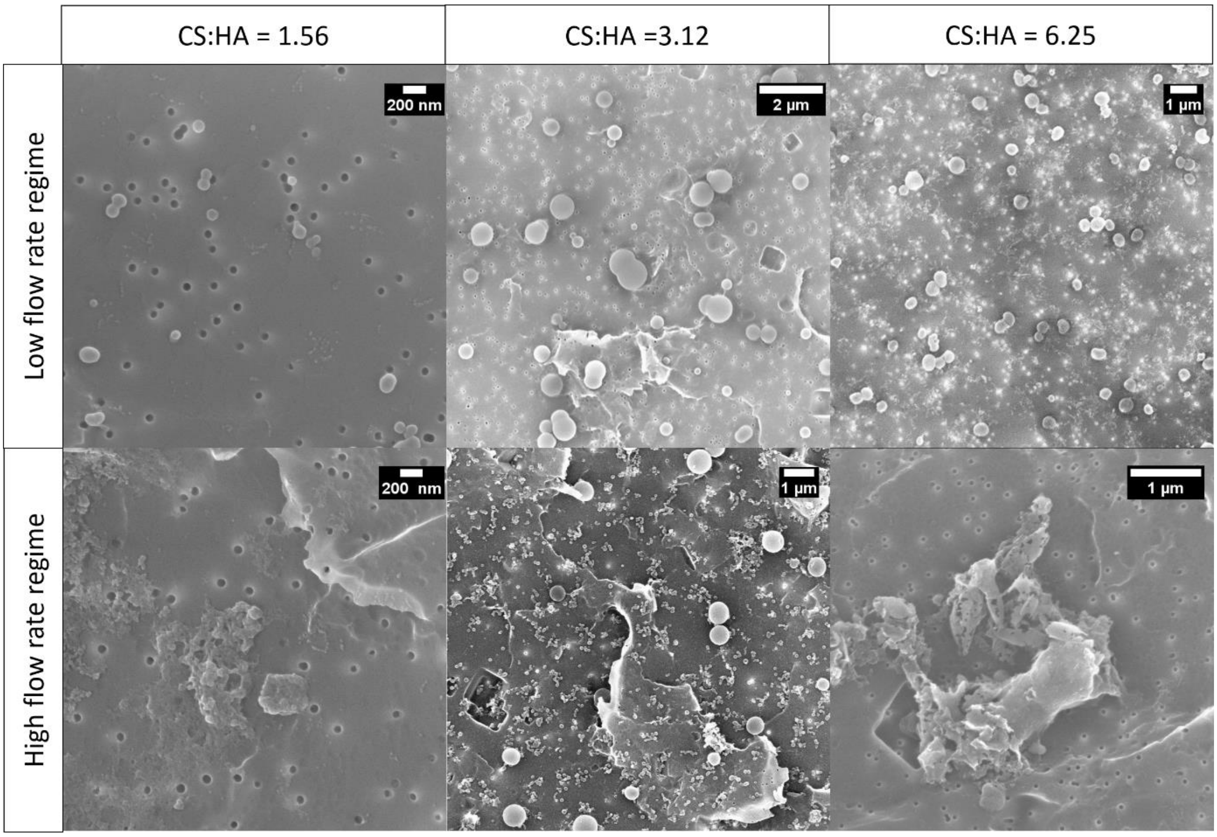


**Figure S4.** Representative SEM images of different CS:HA ratios at $FR^2 = 0.5$.

All the conditions have proved to be suitable to produce NPs except for the conditions related to the lower right SEM image where no NPs are obtained.

**Interpretation of the operational regimes and obtained morphologies**

Size distribution histograms of NPs size at $FR^2 = 0.5$ and CS:HA ratio equal to 1.56 or 3.12 at the *High* and *Low flow rate regimes*.

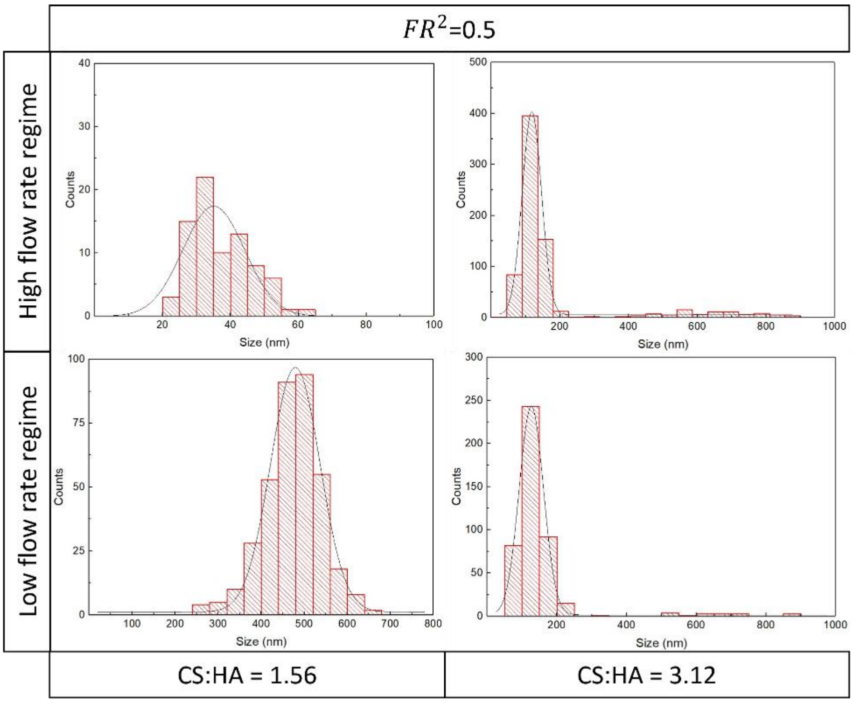


**Figure S5.** Representative histograms and distribution curves of NPs at $FR^2 = 0.5$ and CS:HA ratio equal to 1.56 or 3.12 at the two operational regimes

**Effect of Flow rate ratio at TPP = 0.003% and 0.012%.**

To understand the influence of the crosslinker, two TPP concentrations (0.003 and 0.012 % w/v) have been compared keeping fixed the concentrations of the two polymers (HA = 0.002 % w/v and CS = 0.00625 % w/v). The size of the obtained NPs has been plotted (Figure S6a) as a function of the flow rate ratio (from 0.05 to 0.4) and corresponding representative SEM images have been provided (Figure S6b)

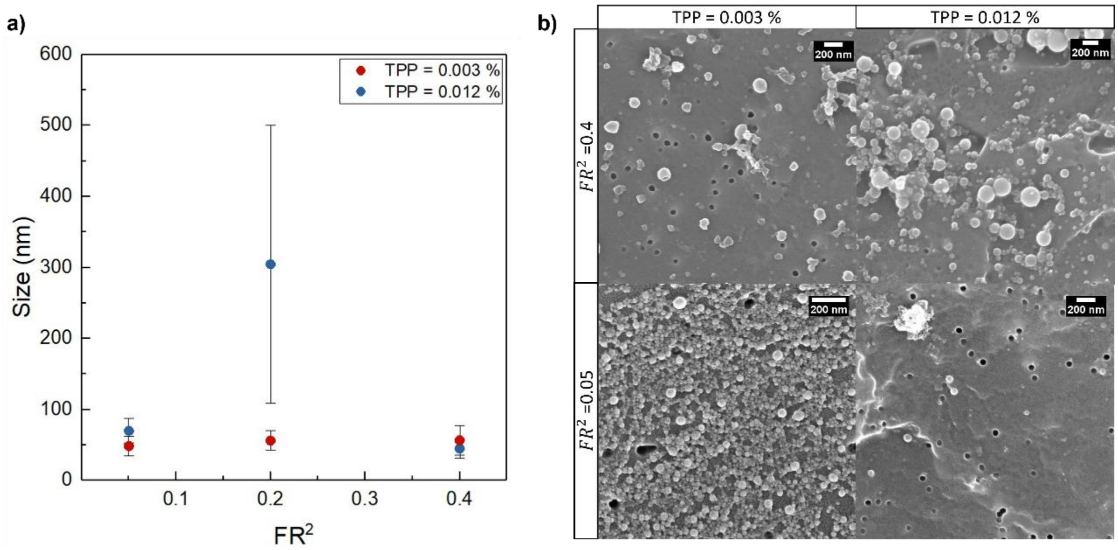


**Figure S6. a)** NPs size as a function of $FR^2$ (0.05, 0.2 and 0.4) at two TPP concentration (0.012 or 0.003% w/v); **b)** SEM images of NPs at two TPP concentrations and two different flow rates.

Figure S6a shows that TPP concentration interferes in coprecipitation process. At TPP = 0.003%, size and polydispersity are very similar regardless the flow rate ratio. On the contrary, at TPP = 0.012% and $FR^2$ = 0.2, NPs are more polydisperse, while, at $FR^2$ = 0.4 and 0.05, they lead to coprecipitates with size and polydispersity comparable to the one obtained at TPP = 0.003%. SEM images in Figure S6b confirm that morphologies are influenced by the flow rates, with size and polydispersity growing proportionally with $FR^2$ and TPP.

**Operational regimes and obtained morphologies.**

Representative architectures at different FR² and CS/HA polymer ratio.

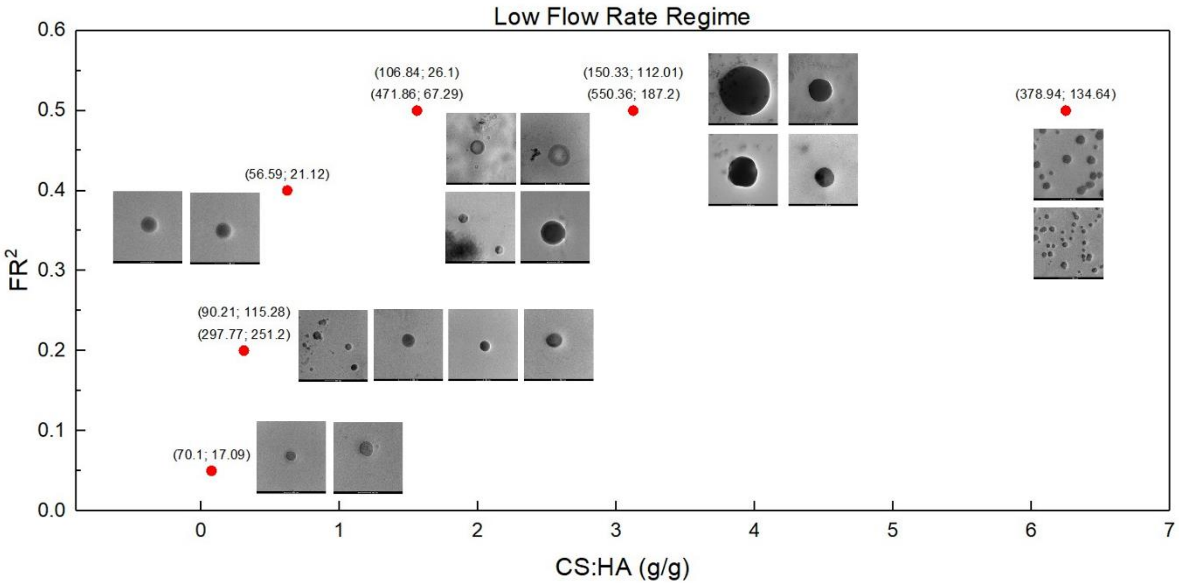


**Figure S7.** Visual plot displaying morphology of NPs at *Low flow rate regime* as a function of FR² and CS:HA ratio. Average diameter and its standard deviation are given in brackets for each trial displayed.

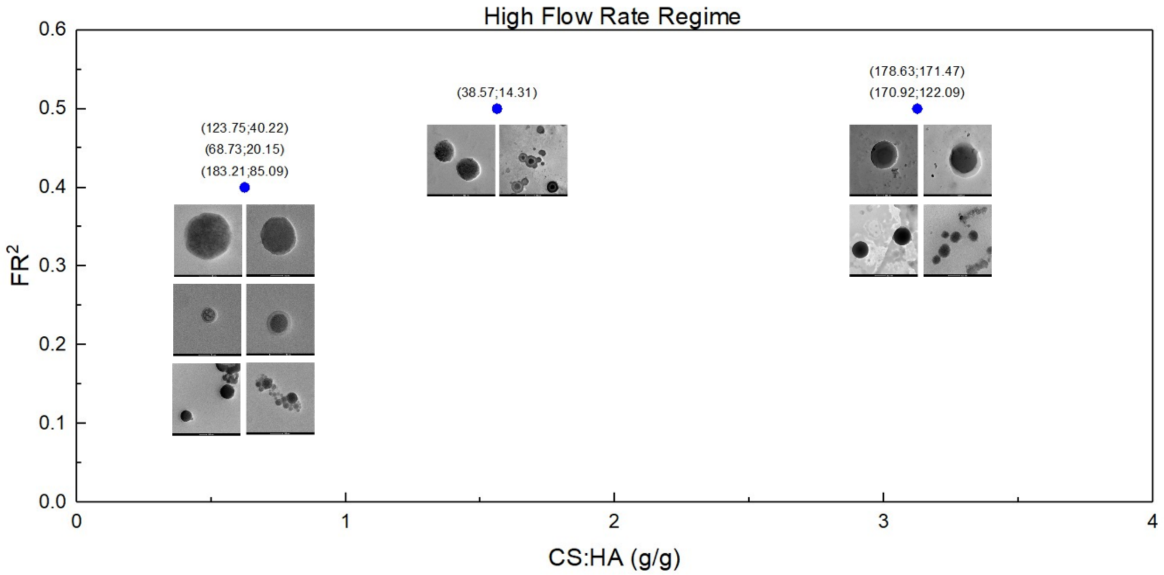


**Figure S8.** Visual plot displaying morphology of NPs at *High flow rate regime* as a function of FR² and CS:HA ratio. Average diameter and its standard deviation are given in brackets for each trial displayed .

**Reynolds numbers**

Numerical estimates of Reynolds numbers within the chip with increasing CS concentration.

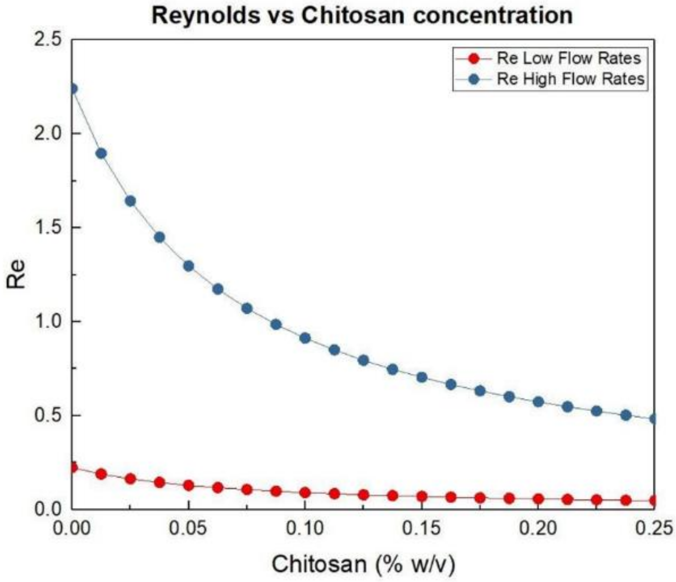


**Figure S9.** Reynolds number as a function of the chitosan concentration at *High and Low flow rate regime*.

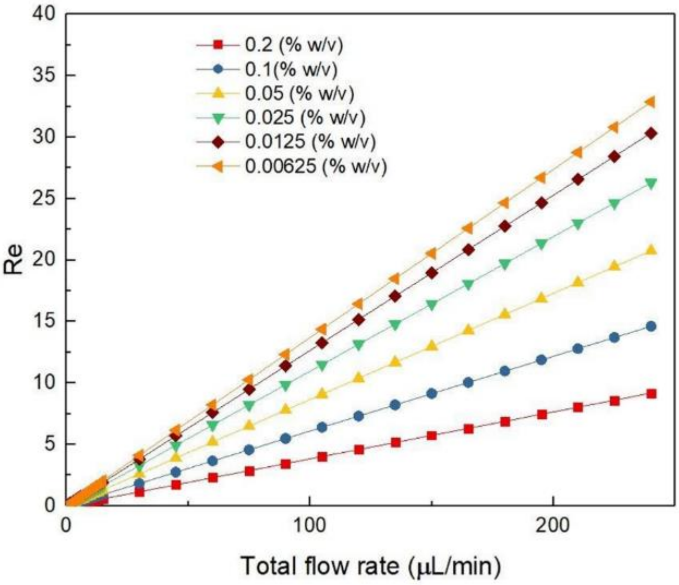


**Figure S10.** Reynolds number as a function of the total flow rate at different chitosan concentrations.

**Gd-DTPA encapsulation.**

The experimental campaign for Gd-DTPA loaded NPs is shown in Table S2 while data regarding the encapsulation efficiency of the CA and the enhancement of longitudinal relaxivity explained through the Hydrodenticity concept are presented in Tables S3 and S4.

**Table S2.** Parameters tested during the experimental campaign after the addition of Gd-DTPA.

| Test # | HA (%w/v) | TPP (%w/v) | CS (%w/v) | Gd-DTPA (%w/v) | Flow rate middle channel (μL/min) | Flow rate side channel (μL/min) | Flow Rate Ratio ($FR^2$) | CS:HA (g/g) |
|---|---|---|---|---|---|---|---|---|
| 1 | 0.008 | 0.012 | 0.1 | 0.1 | 3 | 6 | 0.5 | 3.12 |
| 2 | 0.008 | 0.012 | 0.1 | 0.1 | 3 | 6 | 0.5 | 3.12 |
| 3 | 0.002 | 0.003 | 0.05 | 0.1 | 3 | 6 | 0.5 | 6.25 |
| 4 | 0.002 | 0.003 | 0.05 | 0.1 | 3 | 6 | 0.5 | 6.25 |
| 5 | 0.008 | 0.012 | 0.1 | 0.1 | 3 | 6 | 0.5 | 3.12 |
| 6 | 0.008 | 0.012 | 0.1 | 0.1 | 3 | 6 | 0.5 | 3.12 |
| 7 | 0.008 | 0.012 | 0.1 | 0.1 | 3 | 6 | 0.5 | 3.12 |
| 8 | 0.008 | 0.012 | 0.1 | 0.1 | 3 | 6 | 0.5 | 3.12 |
| 9 | 0.008 | 0.012 | 0.1 | 0.1 | 3 | 6 | 0.5 | 3.12 |
| 10 | 0.008 | 0.012 | 0.1 | 0.1 | 3 | 6 | 0.5 | 3.12 |
| 11 | 0.008 | 0.012 | 0.1 | 0.1 | 3 | 6 | 0.5 | 3.12 |
| 12 | 0.008 | 0.012 | 0.1 | 0.1 | 3 | 6 | 0.5 | 3.12 |
| 13 | 0.008 | 0.012 | 0.1 | 0.1 | 3 | 6 | 0.5 | 3.12 |
| 14 | 0.008 | 0.012 | 0.1 | 0.1 | 3 | 6 | 0.5 | 3.12 |

**Table S3.** Calculation of encapsulation efficiency starting by the measurements taken from the ICP-MS.

| | ICP-MS measured value | Measured mg/L(*1000) | Measured (g/L) | Measured (M) | Measured (μM) | Theoretical (μM) | Encapsulated (measured/theoretical)% |
|---|---|---|---|---|---|---|---|
| Gd-loaded NPs | 0.0022 | 2.2252 | 0.0022 | 4.064E-06 | 4.064 | 34 | 11.95 |

**Table S4.** Calculation of relaxation enhancement starting by the measurements taken from the the benchtop relaxometer.

| | [Gd] (μM) | [Gd] (mM) | $T_1$ (ms) | $T_1$(s) | $\frac{1}{T_1}$(s) | $T_{1\_0}$(s) | $T_{1\_0}$(ms) | $\frac{1}{T_{1\_0}}$(s) | $r_1$ ($s^{-1}mM^{-1}$) | $r_1$ enhancement (n fold) | $\frac{1}{T_1}$ enhancement (%) |
|---|---|---|---|---|---|---|---|---|---|---|---|
| Gd-loaded NPs | 4.06 | 0.004 | 3280 | 3.28 | 0.30 | 3850 | 3.85 | 0.26 | 11.12 | 3.86 | 12.32 |
| Free Gd | 4.06 | 0.004 | 3684 | 3.68 | 0.27 | 3850 | 3.85 | 0.26 | 2.88 | | |

**Fluorophore encapsulation.**

Atto-488 calibration curve used to estimate dye concentration starting from signal intensity detected through spectrofluorometer measurements.

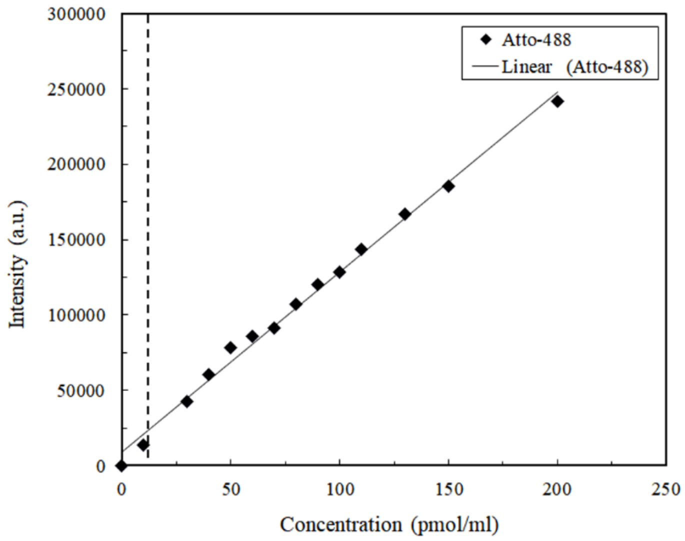


**Figure S11.** Spectrofluorometer calibration curve for Atto-488 in the range 0-200 pmol/mL. Dotted line indicates the measured concentration of Atto-488 encapsualted within the NPs (equal to 7 pmol/ml).

**MTT assay.**

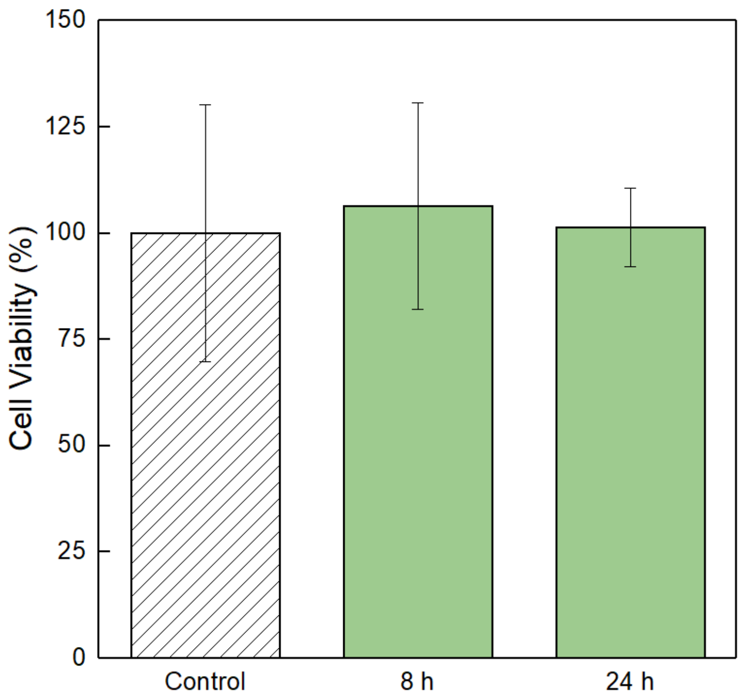


**Figure S12.** In vitro cytotoxicity of CS-HA NPs in contact with U87 MG cells at two different time points. NPs have been produced using the best conditions found out during the experimental campaign (High flow rate regime ( 6 – 3 – 6 μL/min), HA = 0.008 %w/v, CS = 0.1 %w/v, TPP = 0.012 %w/v).
